# Sustainable hydrogen production from water using tandem dye-sensitized photoelectrochemical cells

**DOI:** 10.1186/s40580-021-00257-8

**Published:** 2021-03-02

**Authors:** Benjamin D. Sherman, Nelli Klinova McMillan, Debora Willinger, Gyu Leem

**Affiliations:** 1 Department of Chemistry and Biochemistry, Texas Christian University, Campus Box 298860, Fort Worth, TX 76129 USA; 2 Department of Chemistry, State University of New York College of Environmental Science and Forestry, 1 Forestry Drive, Syracuse, NY 13210 USA; 3 The Michael M. Szwarc Polymer Research Institute, 1 Forestry Drive, Syracuse, NY 13210 USA

**Keywords:** Tandem photoelectrochemical cell, Dye-sensitized photoelectrodes, Water splitting, Solar photocatalysis, Solar fuels, Hydrogen evolution, Solar energy

## Abstract

If generated from water using renewable energy, hydrogen could serve as a carbon-zero, environmentally benign fuel to meet the needs of modern society. Photoelectrochemical cells integrate the absorption and conversion of solar energy and chemical catalysis for the generation of high value products. Tandem photoelectrochemical devices have demonstrated impressive solar-to-hydrogen conversion efficiencies but have not become economically relevant due to high production cost. Dye-sensitized solar cells, those based on a monolayer of molecular dye adsorbed to a high surface area, optically transparent semiconductor electrode, offer a possible route to realizing tandem photochemical systems for H_2_ production by water photolysis with lower overall material and processing costs. This review addresses the design and materials important to the development of tandem dye-sensitized photoelectrochemical cells for solar H_2_ production and highlights current published reports detailing systems capable of spontaneous H_2_ formation from water using only dye-sensitized interfaces for light capture.

## Introduction

Global progress made in medicine, technology, and society since the mid-eighteenth century, but especially in the last 100 years, owes to the use of fossilized carbon fuels to power human industry. While coal, petroleum, and natural gas make ideal fuel stuffs—energy dense, stable, readily transportable—the scientific community has long warned that the re-introduction of sequestered carbon to the active geologic cycle will have profound and detrimental effects on the Earth-climate system [1]. While short-term economic gains have outweighed the immediate and long-term costs of relying on fossil fuels to this point in human history, the growing strength of climate disruptions, and the economic and humanitarian toll thereof [2], might move popular and political perspectives (and policies) toward the complete reliance on sustainable and renewable energy sources. If produced from the decomposition of water, and specifically water sources in contact with the atmosphere, hydrogen gas presents and ideal renewable fuel that could supplant carbon-based fuels for a variety of industrial and economic uses and its combustion product (H_2_O) would not contribute to increased greenhouse effect in the atmosphere.

To realize the potential environmental benefits of hydrogen fuel requires its renewable generation and currently industrial H_2_ production comes from natural gas reformation. This unfortunately creates a carbon footprint for what should be a carbon free fuel cycle. Hydrogen formed from water electrolysis would create no carbon emissions assuming that the electricity to drive the electrolysis comes from renewable generation—e.g., wind, hydroelectric, geothermal, or solar. This approach is not technology limited and with the development of favorable market conditions and/or implementation of environmentally conscious public policies, could become a viable path to supplying H_2_ fuel. Regardless of how the energy is sourced, the decomposition of water to H_2_ and O_2_ products (Eq. 1) is both thermodynamically and kinetically challenging, given the strength of the O–H bond and the need to transfer 4 e^–^ for each molecule of O_2_ formed in the process. A promising approach to overcome these challenges is to integrate energy capture and conversion with catalysis in a single chemical system; a tandem photoelectrochemical cell (PEC) for H_2_ production does just this and offers, at a system level, the means to achieve the highest possible efficiency for using solar energy to form H_2_ from water.

1 $$2{\text{H}}_{2} {\text{O}}\left( l \right) \to 2{\text{H}}_{2} \left( g \right) + {\text{O}}_{2} \left( g \right)\,\Delta G^{^\circ } = 474.2 kJ$$

In a landmark study, Khaselev and Turner demonstrated the promise of using a tandem junction system for H_2_ production from water splitting, reporting 12.4% light-to-hydrogen efficiency (~ 11 sun illumination intensity) using main group III-V semiconductor light absorbers [3]. Adjustment in the band gap and modification of the interfacial layers has resulted in improved solar-to-hydrogen efficiencies of similar monolithic tandem III-V PECs [4], with the highest currently reported efficiency of 19% [5]. A triple junction solar cell comprised of III-V semiconductor materials coupled to an optimized system of water electrolyzers has achieved an impressive solar-to-hydrogen (STH) energy conversion of 30% [6]. While proving the effectiveness of harnessing solar energy for H_2_ production by the photolysis of water, the high production cost of these light absorbing materials has limited their economic viability in current market conditions [7].

Since first reported [8], dye-sensitized solar cells (DSSCs) have held promise as a lower cost alternative photovoltaic technology compared to those based on pure, crystalline semiconductor light absorbers [9, 10] and the same types of dye-sensitized electrodes can be applied to photoelectrocatalytic applications [11]. The advantages of a dye-sensitized photoelectrode include facile production and processing using sol–gel oxide pastes to establish the mesoporous surface and the innate ability to adapt the light absorption and surface redox properties by the adsorption of different dye/catalyst species. This latter feature makes dye-sensitized photoelectrodes especially well purposed to the development of tandem photochemical cells because optimization requires achieving a balance of equal photon flux absorbed at each light active surface, while also tuning the redox levels to have sufficient potential to carry out the desired half reactions within the cell [12].

Specifically with regard to the water oxidation half reaction, dye-sensitized photoelectrochemical cells (DSPECs) have gone from first demonstration [13], to achieving 1 mA cm^−2^ current densities [14], as well as prolonged stability in photocurrent generation [15–17] over just the last ten years. Progress in the design and development of DSPEC photoanodes is available in several other informative review articles [18–22]. A universal attribute of all DSPEC photoanodes for water oxidation is that an applied bias is required to sustain anodic photocurrent and no single junction DSPEC has been shown to split water to O_2_ and H_2_ using only the energy of incident photons. To achieve the overall photolysis of water without any additional electrical energy assistance requires use of a tandem DSPEC where a second dye-sensitized photoanode or photocathode in the system absorbs and converts the energy of a second photon for each electron passed in the cell. Only in the last five years have such tandem DSPECs been reported in the literature and this review will cover the milestone developments and ongoing progress toward the development of tandem dye-sensitized photoelectrochemical cells for spontaneous light driven H_2_ formation from water.

## Efficiency of single vs. tandem junction solar cells

The reason no single junction DSPEC has achieved overall water splitting without assisting electrical bias rests in the energy required to split water (1.23 eV), the need to generate overpotential to drive equilibrium toward the H_2_/O_2_ products and avoid non-productive charge recombination in the system (~ 0.5 eV), and the energy cost of unavoidable internal losses during the conversion of light to electrical energy (~ 0.6 eV). Taken together, a single junction device would require photons with energies of ~ 2.3 eV or higher (< 540 nm) [23]. This precludes the yellow to red portion of the visible spectrum, and though green to blue photons contain sufficient energy, kinetic challenges in the systems studied require additional electrical bias to sustain forward electron transfer and sustain the formation of O_2_ and H_2_ products. Under ideal circumstances and assuming parallel physical behavior to a semiconductor absorber, a DSPEC that used the energy of one photon per each the four electrons transferred in Eq. 1 constructed with a dye absorbing 2.6 eV photons (477 nm) would give the maximum possible solar-to-hydrogen (STH) efficiency of 4.5% for a single junction system [23]. Splitting the energy burden for driving overall water splitting between two light absorbing junctions, however, can achieve a theoretical max STH efficiency of 27% [24]. This assessment gives optimum absorption thresholds of 720 nm for the short wavelength and 1120 nm for the long wavelength junctions and allows for overall loss of 0.8 eV per photon.

**Fig. 1:**
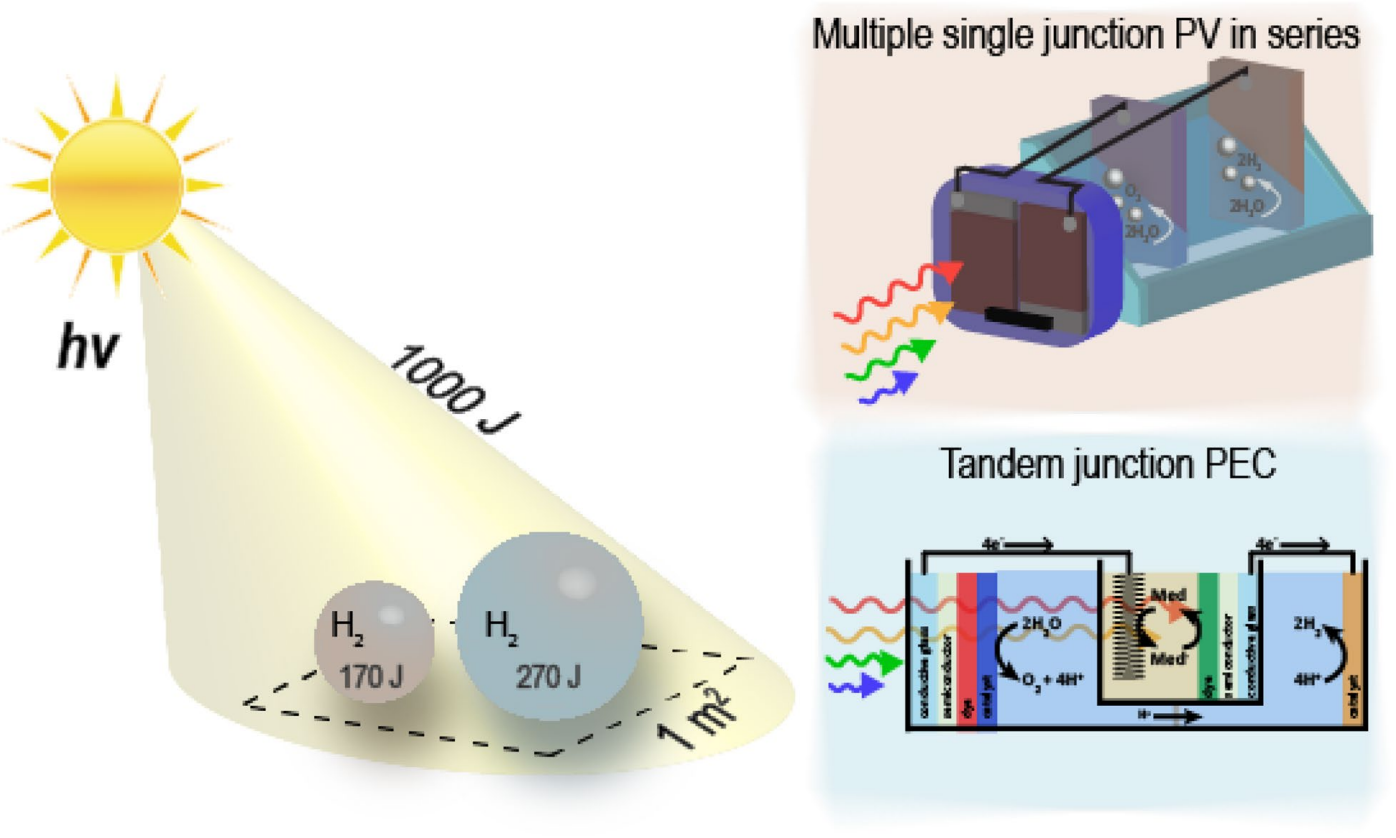
Comparative optimal energy conversion efficiency for AM 1.5 G solar illumination integrated over a 1 s timescale for a single junction (red shaded sphere) or tandem junction (blue shaded sphere) photoelectrochemical cell. The right panels provide example device architecture for a single junction (no change to overall optimum efficiency for multiple single junction devices mounted in parallel to the light source) and tandem DSPEC-DSSC water splitting cell

Several unavoidable loss mechanisms govern the maximum possible STH efficiency of a single, tandem, or other multi-junction type photochemical cell: (1) photons with lower energy than the band gap or HOMO–LUMO gap (U_g_) of the light absorber cannot be converted (incomplete absorption); (2) the excess energy of photons with energy greater than U_g_ is lost as heat during relaxation to the first excited state (thermalization); (3) some fraction of the excited state energy is lost upon conversion to electric or chemical potential energy (overpotential); and (4) some degree, usually minimal, of excited states formed will undergo emission (radiative recombination) [25–27]. Optimum conversion efficiency occurs when these losses sum to ~ 0.3–0.4 eV per photon absorbed, but the overpotential requirements of the catalysts for O_2_ and H_2_ production increase the practical limits to ~ 0.8 eV per photon [24]. Assuming these optimum conditions gives a maximum STH efficiency of 27% for a tandem cell vs. 17% for a single junction cell [24]. Figure 1 illustrates this difference in yield of H_2_ product, in this case based on AM 1.5 illumination integrated over a 1 s time scale and also presents an important point regarding tandem devices—namely that a true tandem system must integrate the photoelectrodes in a stacked (one in front of the other) configuration. This is implicit to realizing the advantage in STH efficiency, though some systems may include multiple photoelectrodes wired in series, if these are not positioned optically in series (i.e., stacked and not side-by-side), the maximum possible STH efficiency remains the same as that of a single threshold device [24].

## Design, critical components, and performance metrics for a tandem DSPEC

### Cell architecture

With the key point that any tandem system must contain the light absorbing electrodes optically stacked in series, there still exist several possible device architectures. This review specifically focuses on tandem dye-sensitized photoelectrodes wired in series, either composed of a photoanode and photocathode or with two photoanodes and two dark cathodes. In the latter case, this tandem configuration can be thought of as a DSPEC wired in series and optically in series with a DSSC (DSPEC-DSSC) and is shown in the lower right of Fig. 1. While this review focuses on DSPEC tandem architectures, several other distinct designs for tandem water splitting PECs (photoelectrochemical cells) have been reported and will be briefly discussed below. Other authors have addressed these systems in more detail [28–30].

As noted in the introduction, PEC-PV (photovoltaic) tandem cells, especially those using main group III-V semiconductors have achieved high STH efficiencies though with substantial material and production cost [3–5]. In these systems, one of the III-V semiconductor surfaces is in contact with aqueous electrolyte (establishing one half of the PEC component). Sufficient photovoltage can be generated by placing two or more PV components in series and connecting the array to an external water electrolyzer or embedding the PV layer with electrolyzer electrodes. Examples exist using triple junction amorphous silicon [31, 32], CIGS (CuIn_x_Ga_1-x_Se_2_) [33], and perovskite [34] light absorbing PV elements. While these systems incorporate multiple light absorbing junctions in electrical series, because each junction absorbs identical portions of the solar spectrum and/or the junctions are mounted side-by-side and not in a stacked configuration, the best possible STH efficiency is identical to an ideal single junction system [24]. To realize the higher efficiency ceiling of a true tandem system requires stacked light absorbing layers that target distinct regions of the solar spectrum.

In pursuit of realizing the higher possible efficiencies of a tandem solar-to-fuel photochemical cell but with lower cost and more easily fabricated materials has led researchers to the use of oxide semiconductors such as TiO_2_, WO_3_, Fe_2_O_3_, and BiVO_4_. The simplest configuration of a tandem PEC consists of a photoanode, incorporating one of the oxides just mentioned, wired to a photocathode with electrolyte solution completing the circuit. Such p/n-PEC tandem cells have been reported though in general these systems achieve modest STH efficiencies due to high band gap energies of the photoanode and/or poor charge transport properties of the photocathode. Some selected examples include those using a TiO_2_ photoanode combined with a CaF_2_O_4_ photocathode (STH =  < 0.1% (est.), illuminated side-by-side, not stacked), [35] a Cu–Ti–O photocathode (STH = 0.3%), [36] and a TiO_2_/Si nanostructured surface (STH = 0.12%) [37]. Efforts to expand photoanode absorbance to the visible motivated studies using Fe_2_O_3_ or WO_3_ photoanodes with a GaInP_2_ photocathode [38] and a BiVO_4_ photoanode/Cu_2_O photocathode tandem cell achieving an STH efficiency of 0.5% [39].

The use of similar oxide semiconductors at the photoanode has resulted in higher solar to hydrogen conversion efficiencies when incorporated in a separate tandem device configuration—that consisting of two photoanodes with two dark cathodes wired in series. A diagram of this configuration, which is equivalent to separate PEC and PV components interconnected with the light absorbing electrodes in a stacked configuration, is shown in Fig. 1. Early examples used a p/n-Si PV cell with a TiO_2_ photoanode, [40] and more contemporary work with WO_3_ [41, 42] and Fe_2_O_3_ [42] photoanode based PEC combined with a dye-sensitized solar cell (DSSC) PV demonstrated STH efficiencies of up to 3.1%. The use of a BiVO_4_ sensitized WO_3_ photoanode increases the STH efficiency to 5.7% of this PEC-DSC tandem design [43] and this increased to 7% with optical engineering of the interfacial layers [44].

This review focuses on a similar type of tandem configuration but one that exclusively uses dye-sensitized photoanodes for all the light absorbing interfaces in the system. The DSPEC-DSSC tandem configuration as such enables more flexibility in modifying the threshold wavelength as well as the redox potential generated at each interface. As the discussion above illustrates, only a handful of light absorbing oxide semiconductors have proven effective in PEC-DSSC tandem systems and their light absorbing and band edge potentials inherently limit the possible efficiency for solar-to-fuel tandem cells. Section 4 of this review will present the progress made to date with DSPEC-based tandem cells and the next subsections introduce the important components and performance metrics used in the development of these systems.

### Oxide semiconductor support

While itself transparent to visible light, the semiconductor electrode surface in a tandem DSPEC must facilitate the generation of charge carriers (electrons for n-type or holes for p-type) upon light illumination for collection at the back contact while the complementary charge (holes for n-type, electrons for p-type) participate in heterogeneous transfer at the oxide/electrolyte surface. The DSPEC oxide semiconductor must be stable in aqueous solution, optically transparent in the visible region, and possess conduction band/valence band energies that can facilitate charge transfer from the surface-bound dye excited state. Most commonly, TiO_2_ or SnO_2_ are used for photoanode and NiO for photocathode electrodes constructed for DSPEC studies [21, 45].

**Fig. 2:**
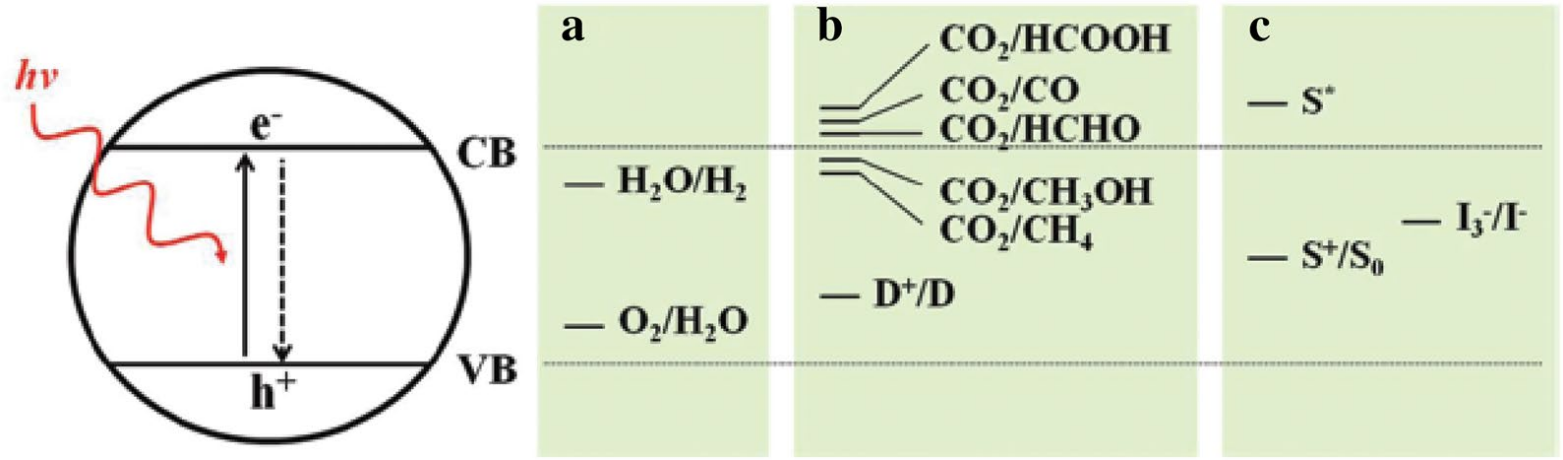
Schematic illustration of TiO_2_ photocatalysis and redox potentials for (**a**) water splitting, (**b**) CO_2_ reduction, and (**c**) DSSCs. CB, conduction band; VB, valence band; D, donor; S, photosensitizer. Reprinted with permission from He et al. [47]. (Copyright 2012 American Chemical Society)

Most photoanodes used for DSSCs and DSPECs rely on TiO_2_ to form the high surface area base support due to its more positive conduction band potential (E_cb_ = − 0.1 V vs NHE at pH = 0), facile synthesis, and stability in both non-aqueous and aqueous phases [46]. Figure 2 shows the band structure of TiO_2_ and relative redox potentials for water splitting, CO_2_ reduction, and redox mediators commonly used in DSSCs [47]. Note that the photoexcited electrons occupying conduction band states in TiO_2_ have sufficient energy to drive H_2_ production when coupled with a suitable catalyst [48].

Meyer and coworkers have demonstrated the remarkable performance for water splitting in a DSPEC with SnO_2_/TiO_2_ core–shell nanostructured DSPEC photoanodes [49]. This enhanced efficiency comes from the use of a SnO_2_ core. SnO_2_ has a conduction band (E_CB_ ~ 0.28 V vs NHE) more positive than that of TiO_2_. Charge recombination between the photoanode and the chromophore or photocatalyst significantly affects the photocatalytic efficiency for water splitting or hydrogen production in a DSPEC system. The SnO_2_/TiO_2_ core/shell network can suppress back electron transfer through the oxide interface between inner SnO_2_ and outer shell TiO_2_ due to the ~ 0.4 V offset in E_CB_ between metal oxides. Sherman et al*.* reported that the photocurrent of a SnO_2_/TiO_2_ core–shell structured DSPEC photoanode showed a six-fold increase, up to 0.85 mA cm^−2^, under identical conditions compared with a TiO_2_ electrode in an otherwise identical DSPEC [50]. Wee et al*.* have highlighted the suppression of back electron transfer at the SnO_2_/TiO_2_ core/shell electrode surface by transient absorption–time and photocurrent–time traces, again compared against mesoporous nanostructured TiO_2_ electrode in contact with aqueous solution [51].

**Fig. 3:**
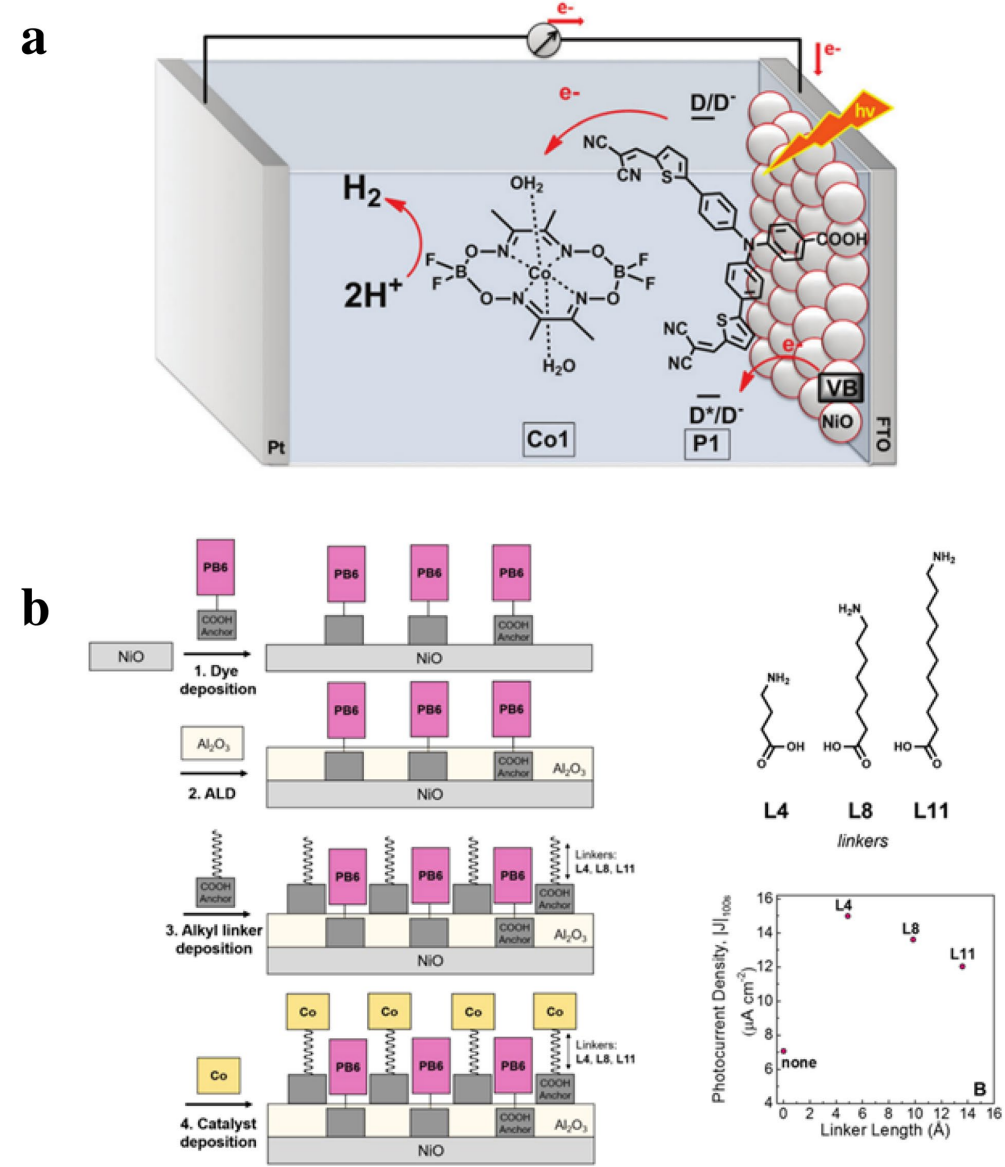
**a** A NiO-based photocathode containing surface bound organic sensitizer (P1), and soluble Co-based proton reduction catalyst (Co1) [53]. **b** NiO photocathode architectures prepared using alkyl-linked catalysts and photocurrent density depending on the length of linkers. PB6: push–pull organic sensitizer, Co: proton reduction catalyst, L: the alkyl linkers [57]. Reproduced from Li et al. [53] with the permission of the Royal Society of Chemistry and Materna et al. [57] with permission from the American Chemical Society, source material available at https://pubs.acs.org/doi/full/10.1021/acsami.0c05228

In DSSCs and DSPECs, NiO has been extensively studied as a p-type oxide due to its easy synthesis, transparency, and appropriate valence band potential (ca. 0.4 V vs NHE in pH 6.8 phosphate buffer) [52]. Sun and co-workers first reported a photocathode based on a D-pi-A structured sensitizer immobilized on NiO (Fig. 3a) [53]. The HOMO of the organic dye is more positive than the valence band of NiO. Therefore, electrons can easily transfer from E_VB_ to photoexcited organic P1 dye. However, it was found that the photocurrent decay observed was due to the decomposition and/or degradation of the catalyst on the NiO electrode surface. Moreover, NiO has a low charge mobility, unfavorable interfacial electron transfer, and shows fast charge recombination [21, 46]. To overcome these barriers, NiO and chromophore structures have been chemically modified leading to more controlled interfacial dynamics [54–56]. For example, the dye containing –COOH moieties was anchored to mesoporous NiO photocathodes followed by coating with Al_2_O_3_ onto the surface bound dye, and the proton reduction catalyst featuring alkyl linkers with different chain length was immobilized on the Al_2_O_3_ coated NiO electrode (Fig. 3b) [57]. An insulating material, Al_2_O_3_, served to encase the surface-bound dye on the NiO electrode and stabilize the –COOH anchoring moiety. Electron transfer kinetics can be controlled by the distance between the dye and catalyst with varying chain length linkers, L4, L8, or L11. The hydrophobic alkyl linkers of the catalyst and Al_2_O_3_ insulating layer for the dye help slow down charge recombination between the organic dye and NiO. As a result, the high photocurrent density was shown with shorter alkyl linkers and the Al_2_O_3_ layer. On the basis of these results, to improve photocatalytic activity in NiO-based photocathodes, surface binding and stabilization of the dye and catalyst are important aspects to consider in the design and fabrication of NiO based photocathodes for use in tandem DSPEC systems.

### Chromophores

**Fig. 4:**
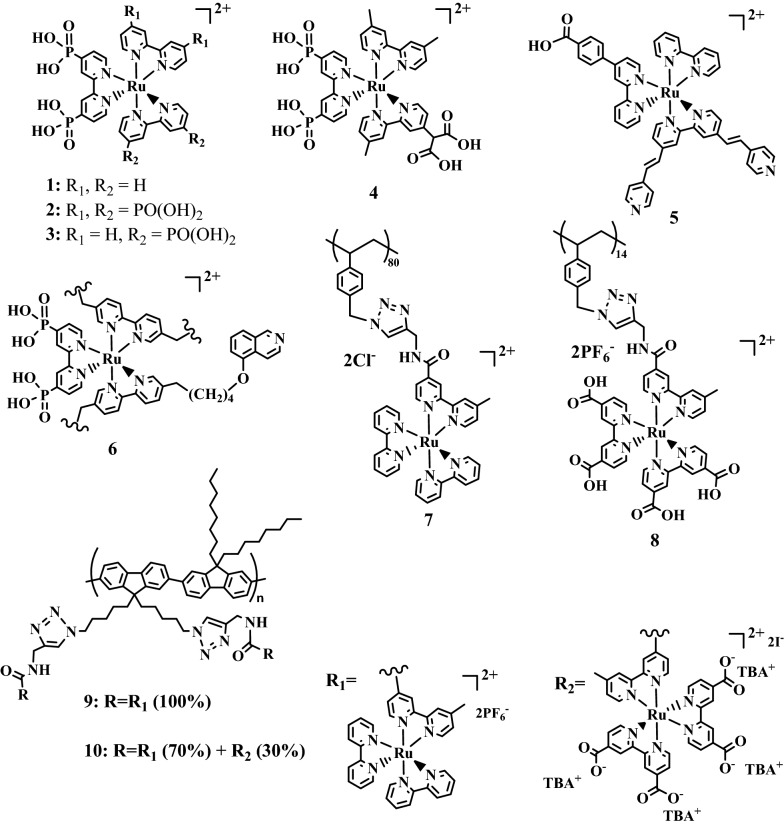
Examples of ruthenium polypyridyl chromophores **1**, [60] **2**, [63] **3**, [61, 65] **4**, [13] **5**, [64] **6**, [12] **7**, [66] **8**, [67] **9**, [68] and **10** [68] used in DSPEC related studies. *TBA*^*+*^ tetrabutylammonium

Molecular chromophores including organometallic dyes, metal-free chromophores, and polymer-based chromophores have been studied in DSSCs, DSPECs, and tandem DPSECs. Especially, Ru(II)-based chromophores have been extensively investigated over the last several decades as light absorbers due to their long-lived excited state lifetimes, high absorptivity in the visible region of λ > 450 nm, and a high oxidation potential [58, 59]. A ruthenium *tris*-bipyridine (Rubpy) containing a phosphonated bipyridine ligand ([Ru(4,4′-H_2_O_3_P-bpy)(bpy)_2_]^2+^, **1**) can be covalently anchored on oxide semiconductor surfaces (e.g., TiO_2_, SnO_2_@TiO_2_ core–shell, NiO) [13, 59–65]. Sheridan et al*.* reported light driven water splitting using the **1** chromophore dye on a SnO_2_@TiO_2_ core–shell electrode coupled to a water oxidation catalyst and electron-transfer mediator (Fig. 4) [60]. Rubpy modified with bidentate carboxylates (**4**) served as both a sensitizer and a molecular bridge as reported by Youngblood et al. [13] Structures **2** and **3** shown in Fig. 4 contain multiple phosphonic acid groups and are designed for assembly on semiconductors [61, 63, 65]. The tetraphosphonated or hexaphosphonated Rubpy derivatives were anchored on the NiO photocathodes by a layer-by-layer method using Zr^4+^ ions that strongly bind to phosphonic acid moieties. Ji et al*.* reported a bifunctional cyclometalated Rubpy chromophore (**5**) with the carboxylic acid anchoring group and the electron-rich moiety (e.g., pyridine) [64]. This cyclometalated Rubpy chromophore was linked between NiO and a cobaloxime catalyst for proton reduction. Interestingly, the Rubpy surface-bound electrode exhibits remarkable stability in aqueous solution and excellent photostability under intense illumination.

Single-site Ru-based chromophores are commonly used in DSSCs and DSPECs. Multichromophoric assemblies can mimic photosynthesis that shows a multi-chromophore antennas effect capable of improving the light-absorbing properties [58]. Recently Leem et al*.* have explored multi-chromophores, polymer-based Ru chromophores in a DSPEC system shown in Fig. 4 (**7**, **8**, **9**, **10**). The carboxylic acid-functionalized Ru complex **8** was assembled in non-conjugated polystyrene backbones (PS-Ru-A) [67]. PS-Ru-A was absorbed onto mesoporous nanostructured TiO_2_ film as a photoanode. Importantly, the photostability of multichromophoric PS-Ru-A on TiO_2_ films was enhanced compared to a single-site Rubpy analogue. Moreover, photophysical properties at PS-Ru-A bound TiO_2_ photoanode reveals an antenna effect, site-to-site energy, and hole transport among the pendant Ru chromophores. Layer-by-Layer (LbL) self-assembly approach allows a cationic polystyrene-based Ru polychromophore without anchoring groups (-COOH or -PO_3_H_2_) to anchor to a TiO_2_ photoanode in the presence of an anionic catalyst by electrostatic interaction. This LbL approach can control the amount of chromophores in the polychromophore. Besides, Leem et al*.* reported an alternative polychromophore in conjugated polymer backbone [68]. The ionic carboxylate-functionalized Ru(II) chromophores were incorporated into a conjugated polymer chain, polyfluorene (PF-Ru) (**9** and **10**). This study clearly demonstrated that coupling conjugated polychromophore featuring Ru(II) assemblies to a semiconductor interface exhibited charge separation between Ru(II) and a semiconductor.

**Fig. 5:**
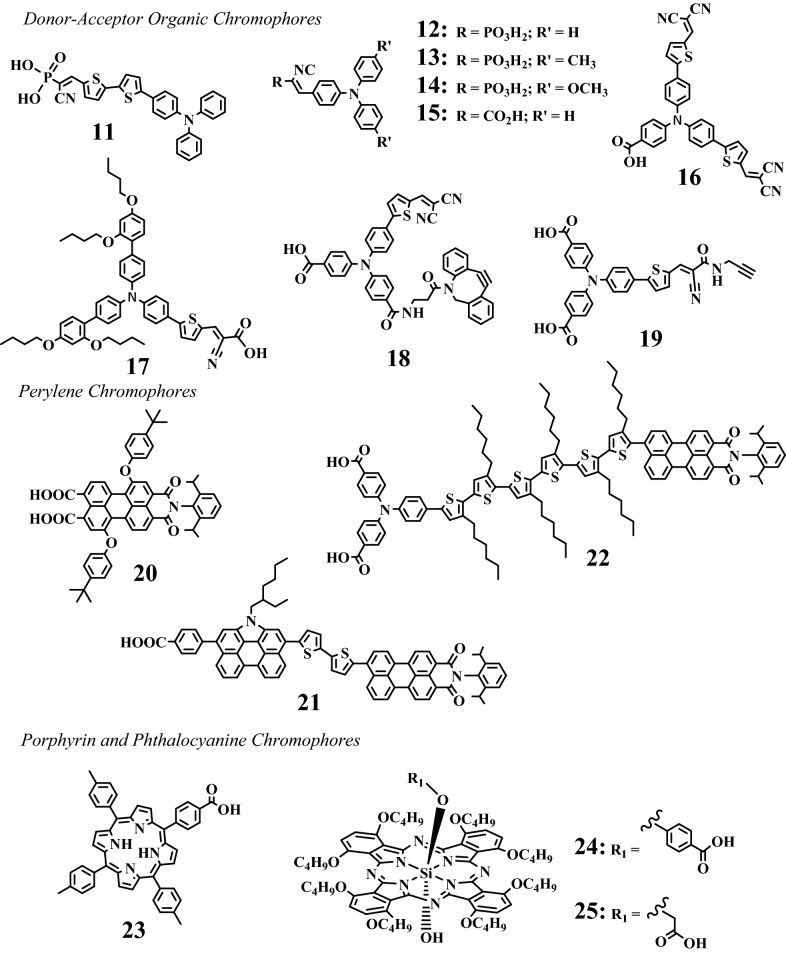
Structures of metal-free chromophores **11**, [51] **12**, [62] **13**, [62] **14**, [62] **15**, [69] **16**, [69] **17**, [70] **18**, [71] **19**, [72] **20**, [73] **21**, [74] **22**, [75] **23**, [12] **24**, [12] and **25** [12] used in selected DSPEC studies

Organic chromophores are integrated for photoelectrochemical water splitting due to strong absorption across the visible spectrum and the ease of modifying their chemical properties through synthesis. Examples of DSPEC and tandem DSPEC devices containing organic chromophores such as donor–acceptor dyes, perylene dyes, and porphyrins are shown in Fig. 5. These organic chromophores provide the required light absorption, redox stability, and an excited-state potential in aqueous phase for water splitting that are competitive with the Rubpy chromophores. For the photocathode, the excited state of the p-type chromophores should be sufficient to inject a hole into the VB of NiO from HOMO of the chromophores, while the excited state of the n-type dyes needs to inject an electron to the CB of TiO_2_ from its LUMO. These interfacial electron-transfer dynamics at the photoelectrodes are important to improving the photocatalytic activity of H_2_ production or water oxidation. Compounds **11**–**19** are examples of donor-π-acceptor (D-π-A) organic chromophores that contain a triphenyl amine unit as electron donor and dicyanovinyl moieties as the electron acceptor [51, 62, 70, 71, 76–79]. Perylene chromophores offer strong reducing power, high fluorescence quantum yield, and excellent molar extinction coefficient [73, 74]. Compounds **21** and **22** are examples of “push–pull” type perylene containing chromophores. Thiophene plays an especially important role of establishing π bridge between the perylene donor and triphenylamine or perylenemonoimide acceptor used in the DSPEC system. Sherman et al*.* reported a tandem DSPEC system incorporating porphyrin and phthalocyanine sensitizers (**23**, **24**, and **25**) [12]. The use of high potential porphyrin chromophores featuring pentafluorophenyl and cyano functional groups are especially well suited to photoanodes for light driven water oxidation.

### Catalysts

Similar methods for surface adsorption of molecular or nanoparticulate catalysts as used for molecular chromophores enable the modification of the photoanode or photocathode surface to support oxygen or hydrogen evolution. Initial work has concentrated on high catalytic turnover but long-term development requires the incorporation of efficient and effective catalysts for water oxidation and H_2_ production that use low cost materials and demonstrate long-term stability. Catalysts used in DSPECs for each half reaction of overall water splitting will be addressed below with a focus on those used in tandem DSPEC systems.

#### Water oxidation catalysis

The first reported DSPEC photoanode for water oxidation used a nanoparticulate IrO_x_•*n*H_2_O catalyst [13]. As one of the most active catalysts for water oxidation, [80] iridium oxide has been widely used in dye-sensitized photochemical applications and is especially useful in these systems because of the ability to functionalize the surface of the colloidal nanoparticles [81–83]. Dye-sensitized photoanodes using Ir-based catalysts do suffer from fast rates of charge recombination [13, 84]. This has led to the pursuit of other catalysts with a focus on the development of molecular catalysts [19, 85] which can be co-adsorbed to the mesoporous oxide surface or incorporated as part of chromophore-catalyst dyads [86].

Of particular interest are the class of [Ru(bda)(L)_2_] (bda = bipyridine dicarboxylic acid, L = neutral donor ligand) catalysts first reported by Sun and coworkers [87, 88]. This particular family of Ru based complexes demonstrate especially high turnover frequencies and require low overpotentials to drive water oxidation catalysis [89, 90]. In particular, [Ru(bda)(isoq)_2_] (isoq = isoquinoline) showed a water oxidation turnover frequency of > 300 s^−1^ which compares to that of the oxygen-evolving complex of Photosystem II of between 100–400 s^−1^ [88]. The observed reactivity of these complexes as water oxidation catalysts (WOCs) has been attributed to the expansion to a seven coordinate species with the incorporation of a water ligand which enables the generation of high oxidation states at relatively negative potentials via proton-coupled electron transfer (PCET) processes. X-ray characterization proved the existence of a seven-coordinate Ru^IV^ dimer reaction intermediate in which water coordinates to the [Ru(bda)(L)_2_] catalyst as the seventh ligand [87]. Introduction of [Ru(bda)(L)_2_] molecular catalysts to TiO_2_ based dye-sensitized photoanodes enabled the first demonstration of > 1 mA cm^−2^ photocurrent densities observed in a DSPEC system [14].

#### Catalyst for hydrogen production

Because most DSPEC studies concentrate on the activity of the photoanode, bulk Pt surfaces are most commonly used as the cathodic catalyst to support H_2_ production. Pt is a good choice of catalyst because of the minimal overpotential required for the onset of proton reduction to hydrogen. The high cost of Pt does make this an undesirable material when considering the long-term development of DSPECs. Nature provides an alternative to Pt in form of hydrogenase enzymes which have been used in bio-hybrid applications [91], including that of a PSII/dye-sensitized tandem PEC [92]. Similar to WOC, molecular complexes offer potential catalysts that can be used either for dark cathodes or as part of a surface modified photocathode, especially those based on Co [93, 94] or Ni [95] metal centers. Emphasis here is on H_2_ production catalysts that have successfully been used in tandem DSPEC systems. Two notable studies include that by Fan et al*.* which used a cobalimine-oxime catalyst for H_2_ production surface immobilized on a sensitized NiO photocathode surface via a pyridine-2,6-dicarboxylic acid anchoring group [96]. Li et al*.* followed up that work with a tandem system using a similar NiO-based photocathode incorporating a [Co(dmgBF_2_)_2_(H_2_O)] (dmgBF_2_ = difluoroboryldimethylglyoximato) complex though in this case modified with phosphonate anchoring groups [76]. Though such photocathodes have not yet been used as part of a tandem DSPEC, Shan et al*.* have reported the use of Ni based molecular H_2_ production catalysts first reported by Dubois and coworkers [97] on NiO based photocathodes [61, 98].

### O_2_/H_2_ quantification and determination of STH

Verifying and quantitating production of the desired O_2_ and H_2_ products in a tandem water splitting solar device represents a key aspect of characterizing a given system. While the observation of sustained photocurrent implies catalytic activity, spurious photocurrent can arise from oxidative decomposition of the chromophore [99] or other non-productive surface reactions. It is therefore paramount that the ultimate characterization metric of the system be that of unambiguously identifying and quantitating the desired reaction products. For solar water splitting in a tandem cell, determination of the solar to hydrogen (STH) efficiency is the accepted and expected means of assessing the activity of the system under study. Equation 2 provides the formula for calculating STH based on photoelectrochemical measurements which takes into account J*,* the steady state photocurrent density under the operating conditions considered; the thermodynamic potential of overall water splitting, 1.23 V vs. NHE; V_bias_, the bias voltage applied to the system if the photocurrent density is not generated spontaneously under illumination; ${\upeta }_{{\mathrm{H}}_{2}\mathrm{O}}$, the Faradaic efficiency for overall water splitting—dependent on ${\upeta }_{{\mathrm{O}}_{2}}$ at the anode and ${\upeta }_{{\mathrm{H}}_{2}}$ at the cathode; and P_in_, the incident light power density which is 100 mW cm^−2^ for 1 sun (AM 1.5) illumination [6] (The units are given in square brackets.)

2 $$\eta_{STH} = \frac{{P_{out} }}{{P_{in} }} = \frac{{\left( {J\left[ {mA cm^{ - 2} } \right] \times \left( {1.23 - V_{bias} } \right)\left[ V \right] \times \eta_{{H_{2} O}} } \right)}}{{P_{in} \left[ {mW cm^{ - 2} } \right]}}$$

The STH efficiency provides an invaluable means of comparing the activity between different systems as well as to the theoretical maximum efficiencies possible. One important point to highlight is that the equation should take into account both the Faradaic efficiency of anodic O_2_ production and cathodic H_2_ formation. Often the Faradaic efficiency of O_2_ production lags that of H_2_ and ignoring this detail can lead to an overestimate of the STH efficiency. Determining either the Faradaic efficiency of O_2_ or H_2_ requires quantifying each species following some operation time of the solar cell. Discussion of the experimental methods for doing so follow below.

#### Determination of O_2_ production

It bears stressing that the observation of photocurrent, whether under an applied bias or under zero bias conditions, does not necessarily equate to the chemical production of O_2_ from water. An essential part of assessing any DSPEC system for water splitting should include both verifying the production of oxygen as well as the determination of the Faradaic efficiency for the O_2_ formed. Determining the Faradaic efficiency requires both the calculation of the total anodic charge passes during an experimental trial under conditions for water oxidation and quantification of the exact amount of O_2_ produced during the same trial. In the simplest form, the Faradaic efficiency is calculated by the ratio of the actual yield of O_2_ observed and the theoretical yield of O_2_ based on the total charge passed (taking into account the four electron oxidation of water to O_2_). Quantitating the actual yield of O_2_ presents greater challenge than the determination of theoretical yield, in large part because of interference from atmospheric oxygen. The unambiguous determination of O_2_ from water is achieved using O^18^ isotopically labeled water combined with mass spectrometry [100]. While O^18^ labeled product O_2_ can rule out non-water oxygen sources and interference from atmospheric O_2_, careful use of hermetically sealed reaction vessels with analysis by gas chromatography can verify the production of oxygen without isotopic labeling so long as control measurement assess the influence, if any, of atmospheric O_2_.

Electrochemistry offers two other methods for detecting and quantitating O_2_ that are especially pertinent to the study of DSPEC water splitting systems. The first is the use of an electrochemical microsensor (Unisense, Denmark) specifically designed to selectively detect and measure oxygen concentrations. Similar to a Clark electrode, these are standalone systems and the sensor is fully contained in a narrow housing with only the probe ending, itself housed in a narrow glass pipet tip or a metal syringe tip, which needs to be in contact with the headspace or solution being measured. These sensors have been successfully used in several studies by the authors [49, 101]. A second electrochemical method for measuring O_2_ is a dual working electrode method (referred to as the collector-generator method) that functions in an analogous manner as a rotating ring-disc electrode but uses two static planar electrodes positioned in close proximity [102]. Based on a method first proposed by Mallouk and co-workers [13], this approach is especially well adapted to study of the fluorine-doped tin oxide (FTO) based photoanodes used in DSPEC studies [103–107]. This approach allows for straight forward determination of Faradaic efficiencies for O_2_ formation and the real-time monitoring of O_2_ generation activity assuming correct conditions are maintained and control experiments carried out [102, 108].

#### Determination of H_2_ production

Similar approaches as used for O_2_ are applicable when detecting and measuring the amount of hydrogen formed during a photochemical measurement. Headspace sampling by gas chromatography with thermal conductivity detector (GC-TCD) is a standard approach used for quantitating H_2_ and does not present as large a challenge as with O_2_ in avoiding apocryphal readings [12, 76]. A similar microsensor is available with H_2_ specific response that allows for real-time monitoring and low detection limits that has proven quite effective in DSPEC related studies [16, 49, 70].

## Reported tandem DSPEC systems

This review focuses on tandem DSPEC systems for solar water splitting. As discussed earlier, semiconductor based tandem cells and semiconductor/DSSC tandem systems have been reported that show robust STH efficiencies but in these cases the ability to tune the absorption characteristics or catalytic activity of a given interface is inherently limited by the material properties of the chosen semiconductor. In theory, exclusively utilizing dye-sensitized photoelectrodes to comprise the tandem system should enable nearly unlimited opportunity to change the properties of each interface, or at least the only limitation being synthetic or structural constraints intrinsic to chemistry. This presents a key justification for pursuing tandem DSPEC systems since it provides a path toward reaching STH efficiencies near the theoretical limits, which is also essential to making solar fuels economically feasible. Despite this potential, only a handful of tandem DSPEC systems have been reported to date. This in part reflects the difficulty in fabricating and studying DSPEC photoelectrodes—requiring endeavors and expertise in materials chemistry, organic synthesis, electrochemistry, inorganic chemistry, photochemistry, and spectroscopy—and also in the key technical challenges of developing electrode interfaces with long-term stability while promoting forward charge transfer and avoiding non-productive charge recombination. Table 1 provides key details and performance metrics for the list of tandem DSPEC water splitting systems reported in the literature to date.

**Table 1:** Key components and performance data for tandem DSPECs for H_2_ production

Tandem Type	Dye/Catalyst^a^	Oxide Support^a^	Photocurrent Density	STH%	Notes	Ref.
n/p-DSPEC	**1/**Ru(pdc)(pic)_3_ **1**/CoHEC	TiO_2_ NiO	12 µA cm^−2^	–	Side-by-side illumination	Fan et al. [96]
n/p-DSPEC	**15**/Ru(pdc)(pic)_3_ **16**/Co(dmgBF_2_)_2_(H_2_O)	TiO_2_ NiO	70 µA cm^−2^	0.05%	${\eta }_{{O}_{2}}$= 55% Side-by-side illumination at 1 sun	Li et al. [76]
DSPEC-DSSC	**23** **24** or **25**	SnO_2_ TiO_2_	20 µAcm^−2^	–	Hydroquinone sacrificial donor	Sherman et al*.* [12]
DSPEC-DSSC	**6**/Ru(bda) **17**	SnO_2_@TiO_2_ TiO_2_	40 µAcm^−2^	0.06%	${\eta }_{{O}_{2}}$= 45% (pH = 9)	Sherman et al*.* [12]
DSPEC-OSC	**1**/Ru(bda) BnDT-FTAZ/ITIC	SnO_2_/TiO_2_ Zn|ITO, MoO_3_-Al	1.24 mAcm^−2^	1.5%		Wang et al. [109]
DSPEC-PV	**1**/Ru(bda) p/n-Si	TiO_2_	0.1 µAcm^−2^	0.1%	${\eta }_{{O}_{2}}$= 79%; ${\eta }_{{H}_{2}}=100\%$	Sheridan et al*.* [89]

^a^Photoanode components listed first, photocathode second for n/p-DSPECs. More-blue absorbing junction listed first, more-red absorbing junction listed second for DSPEC-DSSCs

A survey of Table 1 reveals two types of device architectures encompass all tandem DSPECs, either those composed of a photoanode and photocathode (n/p-DSPEC) or four electrode systems consisting of two n-type photoanodes and two dark cathodes (DSPEC-DSSC). Fan et al*.* were first to report an n/p-DSPEC tandem cell employing a TiO_2_ photoanode with co-adsorbed **1** and Ru(pdc)(pic)_3_ as WOC and a NiO based photocathode with **1** and Co containing H_2_ catalyst (CoHEC) [96]. Importantly, when wired together with zero applied bias between the two photoelectrodes, the tandem system generated a stable photocurrent density of 12 μA cm^−2^ under 1 sun illumination [96]. It should be noted that this result was obtained with both the photoelectrodes receiving 1 sun illumination—this was done in a ‘side-by-side’ configuration and not a stacked ‘tandem’ configuration—and therefore would be limited by the theoretical max efficiency of a single threshold system rather than a true ‘tandem’ photocell (see discussion in part 2). The authors did not carry out O_2_ or H_2_ measurements making a determination of what fraction of the steady-state photocurrent under no applied bias resulted in overall water splitting.

Sun and co-workers followed up their earlier study with a second report of an n/p-DSPEC, this system utilized similar molecular catalysts at the TiO_2_ photoanode and NiO photocathode but relied exclusively on organic triphenylamine based chromophores at each interface (**15** and **16**) [76]. While containing overlapping absorption bands in the blue to UV region, the absorption band of **16** does extend farther to the red (λ_max_ = 481 nm) than **15**. Here again, results are reported with the photoanode and photocathode illuminated in a ‘side-by-side’ type configuration where each receives 100 mW cm^−2^ white light unshaded by the other. This system demonstrated improved performance over the earlier system [96] achieving a steady-state photocurrent of ~ 70 μA cm^−2^ and an overall STH efficiency of 0.05% over a 100 min photolysis period.

A major hurdle to improving the STH performance of p/n-DSPECs lies in realizing higher photocurrent activity from dye sensitized p-type photocathodes which have lagged behind the photoperformance of n-type photoanodes [11, 110]. A tandem DSPEC which consists of separate n-type single junction DSPEC and DSSC elements bypasses the limitations imposed by the p-type interface. The demonstration of such a tandem system by Moore and co-workers used a porphyrin (**23**) sensitized SnO_2_ based DSPEC wired in series with a Si inserted phthalocyanine sensitized (**24** or **25**) TiO_2_ based DSSC to photochemically transform hydroquinone (QH_2_) to H_2_ [12]. While this system did not achieve H_2_ formation from water due to the poor performance of a porphyrin-IrO_x_•*n*H_2_O photoanode pursued at the time (such a construct was later successfully demonstrated [111]), the SnO_2_-based DSPEC alone could not spontaneously carry out the generation of H_2_ from QH_2_. Only with the added photovoltage provided by the DSSC could the overall chemistry proceed, with the two photoanodes in a true tandem stacked configuration. This study showed the promise of a tandem DSPEC in targeting separate portions of the solar spectrum with the porphyrin sensitizer absorbing light out to 650 nm and the phthalocyanine sensitizer used showing a λ_max_ at ~ 780 nm. The use of SnO_2_ at the aqueous photoanode of the DSPEC also has important implications for water splitting applications as it enables the use of high potential chromophores which can provide more overpotential to drive water oxidation but do not have sufficiently reducing excited states to sensitize TiO_2_.

Meyer and co-workers reported a tandem DSPEC-DSSC system that did achieve overall water splitting to H_2_ and O_2_ with the only energy input from light [70]. Two critical innovations led to this achievement—the development and use of SnO_2_@TiO_2_ core–shell oxide interfaces formed by atomic layer deposition which show drastically improved photodynamics compared with SnO_2_ interfaces [112] and the incorporation of [Ru(bda)(L)_2_] type water oxidation catalysts. In this system, light first passed through the DSPEC photoanode where **6** ([ruthenium(5,5′-divinyl-2,2′-bipyridine)_2_(2,2′-bipyridine-4,4′-diylbis(phosphonic acid))]^2+^) absorbs wavelengths shorter than 490 nm before passing on to the DSSC photoanode where the **17** absorbs out to 580 nm. While not the optimal threshold wavelengths for solar water splitting, this system achieved a steady state photocurrent density of ~ 40 μA cm^−2^ leading to an observed STH efficiency of 0.06%. This study marked the first instance where only n-type dye-sensitized photoanodes were used to carryout unassisted solar water splitting.

Carrying on progress with tandem DSPEC based water splitting, Wang et al*.* recently reported an improved system which achieved a remarkable STH efficiency of 1.5% [109]. In this case, the authors used a solid state organic solar cell (OSC) with BnDT-FTAZ [113] donor and ITIC [114] acceptor polymer layers which shows strong absorbance from 500 to 750 nm in a stacked configuration with a ruthenium(II)(bpy)_2_(2.2′-bipyridine-4,4′-phosphonic acid) (**1**) sensitized SnO_2_@TiO_2_ core–shell photoanode with co-adsorbed [Ru(bda)(4,4′-bipyridine)_2_] WOC. A Pt dark cathode completed the DSPEC component of the tandem cell, and under 1 sun illumination the system achieved a photocurrent density of ~ 1 mA cm^−2^ over a 1 h photolysis period. This improved performance was largely due to the enhanced performance of the DSPEC component as compared to the earlier reported DSPEC-DSSC, though the more-red absorption and modestly higher open circuit voltage of the OSC contributed to the higher observed STH. The remarkable improvement in solar to hydrogen efficiency, representing an order of magnitude increase compared to the previous systems, over such a short time from the first reports of tandem dye-sensitized water splitting systems show the promise of this type of tandem photocell. With continued progress this approach should offer a viable alternative to the semiconductor absorber-based systems.

One other tandem systems incorporating a DSPEC photoanode merits mention though it does not fit in exactly the same vein as the systems described above. The report by Sheridan et al*.* details the use of a monolithic tandem junction comprised of a p/n-Si PV embedded base layer with outer mesoporous TiO_2_ surface with adsorbed **1** and [Ru(bda)(4-O-(CH_2_)_3_-PO_3_H_2_-pyr)_2_] (pyr = pyridine) dye/catalyst monolayer [89]. The p/n-Si PV component provides sufficient bias under illumination to facilitate ~ 100 μA cm^−2^ current density over a 15 min illumination period at 1 sun intensity. Microsensor detection of H_2_ and collector-generator analysis for O_2_ demonstrated the production of each at Faradaic efficiencies of 100 and 79% respectively. Though unreported, the STH efficiency is estimated at 0.1%. Pursuing monolithic tandem DSPEC such as this provides another avenue toward leveraging the adaptable and tunable nature of dye-sensitized surfaces for unbiased solar water splitting.

## Conclusions and outlook

Tandem photochemical cells offer the best opportunity for realizing conversion efficiencies that could ultimately provide a sustainable and alternate means of supplying chemical fuel to society in place of fossilized carbon sources. While the current state-of-the-art dye-sensitized tandem photochemical cells lag in terms of STH efficiency compared to those using semiconductor light absorbing materials, the inherent flexibility in modifying and tailoring a dye-sensitized interface to achieve specific absorption and redox properties could provide the means to push realizable efficiencies further toward the theoretical ceiling. While substantial challenges remain including the need to improve long-term stability, finding ever more active and robust catalysts for the required half reactions of water splitting—especially that of water oxidation—and constructing and controlling interfacial architectures to avoid non-productive charge recombination, tandem DSPEC systems have improved STH efficiencies by a factor of 10 across only a few studies in as many years. With continued progress these systems could offer low-cost and easily fabricated devices for efficient solar fuel production and could have other potential applications such as has been shown recently for the depolymerization of lignin [115].

## Data Availability

Not applicable.
